# Ion channel Piezo1 activation promotes aerobic glycolysis in macrophages

**DOI:** 10.3389/fimmu.2022.976482

**Published:** 2022-09-02

**Authors:** Shaoqiu Leng, Xiaoyu Zhang, Shuwen Wang, Jing Qin, Qiang Liu, Anli Liu, Zi Sheng, Qi Feng, Xiang Hu, Jun Peng

**Affiliations:** ^1^ Department of Hematology, Qilu Hospital, Cheeloo College of Medicine, Shandong University, Jinan, China; ^2^ Shandong Provincial Key Laboratory of Immunohematology, Qilu Hospital, Cheeloo College of Medicine, Shandong University, Jinan, China; ^3^ Advanced Medical Research Institute, Shandong University, Jinan, China

**Keywords:** Piezo1, macrophage, glycolysis, colitis, HIF1 alpha

## Abstract

Altered microenvironmental stiffness is a hallmark of inflammation. It is sensed by the mechanically activated cation channel Piezo1 in macrophages to induce subsequent immune responses. However, the mechanism by which the mechanosensitive signals shape the metabolic status of macrophages and tune immune responses remains unclear. We revealed that Piezo1-deficient macrophages exhibit reduced aerobic glycolysis in resting or liposaccharide (LPS)-stimulated macrophages with impaired LPS-induced secretion of inflammatory cytokines *in vitro*. Additionally, pretreatment with the Piezo1 agonist, Yoda1, or cyclical hydrostatic pressure (CHP) upregulated glycolytic activity and enhanced LPS-induced secretion of inflammatory cytokines. Piezo1-deficient mice were less susceptible to dextran sulfate sodium (DSS)-induced colitis, whereas Yoda1 treatment aggravated colitis. Mechanistically, we found that Piezo1 activation promotes aerobic glycolysis through the Ca^2+^-induced CaMKII-HIF1α axis. Therefore, our study revealed that Piezo1-mediated mechanosensitive signals Piezo1 can enhance aerobic glycolysis and promote the LPS-induced immune response in macrophages.

## Introduction

Macrophages are the first encounters with invading bacteria and initiate immune responses to pathogens delivered through circulation (1–3). The function of macrophages is finely tuned by multiple environmental factors, such as temperature, pH, and oxygen concentration (4–6). Recently, several studies have shown that multiple types of mechanical stresses, including hydrostatic pressure, shear stress, and tensile force, are closely associated with inflammatory responses (7–10). For example, cyclical hydrostatic pressure (CHP) and compressive strain promote the secretion of proinflammatory cytokines (11). The physical properties (roughness, stiffness, porosity, and viscosity) of various biomaterials can also be sensed by macrophages, polarizing them into an inflammatory phenotype (12). These environmental cues may be important for inducing proper immune responses to avoid insufficient or excessive inflammation. However, the underlying mechanisms of mechanosensation-induced immune responses are still not fully understood.

Metabolic reprogramming is critical to the functional state of macrophages. Activation of proinflammatory macrophages results in a metabolic switch from oxidative phosphorylation (OXPHOS) to aerobic glycolysis, similar to the Warburg effect in tumors (13–16). Glycolysis is a metabolic pathway that converts glucose into pyruvate and is controlled by various glycolytic enzymes (17). Inhibition of glycolysis can affect many functions of classically activated macrophages such as phagocytosis, ROS production, and cytokine secretion (18, 19). Aerobic glycolysis supports the energy and nutrition demand of activated macrophages by quickly providing metabolic intermediates for molecule synthesis, although it is an inefficient pathway in terms of ATP production (20). Environmental signals can induce metabolic reprogramming in macrophages (21); however, the effects of physical stimulation on the metabolic status of macrophages remain unclear.

The mechanisms of mechano-induced macrophage activation and the mechanosensitive ion channel, Piezo1 are well-studied. The Piezo1 channel is a mechanosensitive, nonselective cation channel that can be activated by pressure, blood flow-associated shear stress, CHP, the chemical agonist Yoda1, and even by direct lipopolysaccharide (LPS) stimulation or bacterial infections (22–25). Activated Piezo1 converts the applied force into Ca^2+^ influx, which plays a vital role in vascular development, erythrocyte homeostasis, bone remodeling, and heart mechano-chemo transduction (26–28). A few studies have reported that the Piezo1-mediated Ca^2+^ signal in macrophages promotes cytoskeleton remodeling, intracellular organelle trafficking, and a prolonged proinflammatory expression profile (29–34). Considering the association between metabolic status and immune responses, Piezo1 activation might contribute to metabolic reprogramming in macrophages; however, this has not yet been reported.

This study aimed to investigate whether mechanical forces regulate the metabolic status of macrophages by activating Piezo1 using conditionally Piezo1-deficient mice. Our results showed that Piezo1 deficiency contributed to decreased glycolysis, thereby reducing proinflammatory cytokine production by regulating the expression of Ca²⁺/calmodulin-dependent protein kinase II (CaMKII) expression and stability of the hypoxia-inducible factor 1 α (HIF1α) stability. Furthermore, conditionally Piezo1-deficient mice were less susceptible to dextran sulfate sodium (DSS)-induced colitis, whereas Yoda1 treatment aggravated disease progression. Therefore, this study revealed that Piezo1-mediated mechanosensation induces a metabolic switch towards aerobic glycolysis and potentiates the inflammatory responses of macrophages.

## Materials and methods

### Ethics statement

All animal experiments performed in this study were approved by the Animal Ethics Committee of Cheeloo College of Medicine, Shandong University.

### Animals

Mice were maintained in an SPF facility (23°C, 12 h light/dark cycle, 40-60% humidity, food and water *ad libitum*). B6.Cg-*Piezo1*tm2.1Apat/J (JAX Stock No:029213, *Piezo1* ^flox/flox^) mice were purchased from Jackson Laboratory (Bar Harbor, ME, USA). *Csf1r* ^CreERT2^ transgenic mice were designed using Cyagen Biosciences (Santa Clara, CA, USA). *Lyz2* ^cre^ mice were kindly provided by Prof. Qi Xiaopeng, *Piezo1* ^flox/flox^ mice were hybridized with *Csf1r* ^CreERT2^ or *Lyz2* ^cre^ mice to obtain Piezo1 conditional knockout mice. Genotype analysis primers used were as follows:

*Piezo1* ^flox/flox^: P1, 5′-GCC TAG ATT CAC CTG GCT TC-3′; P2, 5′-GCT CTT AAC CAT TGA GCC ATC T-3′;

*Csf1r* ^CreERT2^: P1, 5′-CTG GAG AGG AGA GAC CAG GTG AGA G-3′; P2, 5′-CAT TGA AAC ACG AGA GTT TGG GAC G-3′; P2, 5′-TCTACTTCATCGCATTCCTTGCA-3;′

*Lyz2* ^cre^: P1, 5′-CCC AGA AAT GCC AGA TTA CG-3′; P2, 5′-CTT GGG CTG CCA GAA TTT CTC-3′.

### Cell culture and reagents

To induce bone marrow-derived macrophages (BMDMs), specific genotype mice aged 8-12-week-old were assigned to euthanasia with overdose isoflurane inhalation (concentration of 5%). Continue isoflurane inhalation until 1 minute after breathing stops. Collect the femur and tibia. Flushed bone marrow and red blood cells were removed with lysis buffer and then filtered through a cell strainer. The filtered cells were cultured in a complete DMEM conditioned medium with 50 ng/ml recombinant mouse M-CSF (PeproTech, Rocky Hill, NJ, USA) for BMDM differentiation. Differentiated BMDM were analyzed using flow cytometry. We used an *in vitro* mechanical stress culture model according to the reference for CHP treatment (31). BMDMs were cultured in the cell slide and put into the pressure chamber. The BMDMs were treated under cyclical hydrostatic pressure cycling once per second from 45 mmHg to 60 mmHg with 5% CO2 at 37°C. LPS was purchased from Sigma-Aldrich (St Louis, MO, USA). Yoda1, 2-DG, nigericin, and KN93 were purchased from Selleck Chemicals (Houston, TX, USA). Recombinant mouse IL-4 was purchased from BioLegend (San Diego, CA, USA). For siRNA transfection, BMDMs were plated at a concentration so that cells reached 80% confluence 1 day before transfection. Lipofectamine 3000 transfection agent (Invitrogen, USA) were used according to the manufacturer’s instructions. siRNA sequences were showed in **Supplementary Table 1**.

### Seahorse metabolic assay

BMDMs were plated into XFe96 cell culture microplates (Agilent Technologies, Santa Clara, CA, USA) one day before the assay experiment and incubated overnight at 37°C and 5% CO_2_. On the day of testing, BMDMs were washed with XF RPMI medium and pretreated with this medium supplemented with 1 mM sodium pyruvate, 2 mM l-glutamine, and 10 mM glucose. For the glycolytic rate assay, 5 µM Rot/AA was added to port A and 500 mM 2-DG was added to port B following the recommended 10x concentrations. For the XF Cell Mito Stress Test, 15 µM oligomycin was added to port A, 10 µM FCCP to port B, and 5 µM Rot/AA to port C following the recommended 10x concentrations. The XF Cell Mito Stress Test Kit (Agilent Technologies) and the XF Glycolytic Rate Assay Kit (Agilent Technologies), were used for the seahorse metabolic assay.

### RNA extraction and real-time quantitative polymerase chain reaction

TRIzol reagent (Invitrogen, Waltham, MA, USA) was used to lyse and extract RNA from the cells. mRNA was converted into cDNA using the Prime Script RT reagent kit (Takara Bio Inc., Kusatsu, Shiga, Japan) according to the recommended protocol. The mRNA levels were quantified using TM SYBR Green PCR Kit (Takara Bio Inc.) Data were measured using a Light Cycler 480 System (Roche Applied Science, Penzberg, Germany).

Allele-specific primers are provided in **Supplementary Table 1**.

### Western blotting

Cultured cells were collected and washed with phosphate-buffered saline (PBS). Total or nuclear proteins of different samples were extracted using a protein extraction kit (BestBio Company, Shanghai, China) following the recommended protocol. The concentration was measured using a BCA Protein Assay Kit (Beyotime Biotechnology, Jiangsu, China), and then the denture protein was separated by 10% SDS-PAGE. Proteins were separated using SDS-PAGE (Life Technologies, Carlsbad, CA, USA) and transferred to a polyvinylidene fluoride (PVDF) membrane (MilliporeSigma, Burlington, MA, USA). Antibodies against HIF1α (ab237544), CaMKII (ab52476), and KAT3B/p300 (ab275378) were purchased from Abcam (Cambridge, UK), and phospho-CaMKII (Thr286) was purchased from Cell Signaling Technology (Danvers, MA, USA). HRP-conjugated beta-actin monoclonal (HRP-60008) and secondary antibodies were purchased from Proteintech Group (Chicago, IL, USA). All antibodies were dissolved and preserved according to the manufacturer’s instructions.

### Flow cytometry assay

Cultured or primary mouse cells were resuspended in 1% BSA. Cells were then incubated with anti-CD16/32 antibodies before incubation with flow cytometry antibodies. IgG2b antibodies were used as an isotype control. After washing the antibodies twice with PBS, the fluorescence signal was acquired using a flow cytometer (Beckman Coulter, Brea, CA, USA). The antibodies were purchased from BioLegend.

### Enzyme-linked immunosorbent assay

Cell culture supernatants of mouse sera were collected, and mouse IL-1β, IL-6, and TNF-α levels were measured according to the kit instructions (DAKEWEI, China).

### Immunofluorescence and immunohistochemistry

Cells or tissue sections were fixed in 4% paraformaldehyde for 15 min, washed in PBS, and permeabilized in 0.3% Triton-X in PBS for 10 min. Samples were washed thrice with PBS and then blocked with 5% donkey serum in PBS. Samples were then incubated with primary antibodies at 1:50–1:200 dilution at 4°C overnight and then incubated with Alexa-conjugated secondary antibodies for 1 h at room temperature. A Zeiss LSM 900 laser scanning confocal microscope was used to visualize the sections. For IHC analysis, paraffin sections were dewaxed with xylene and gradient ethanol. The sections were antigen-repaired with citric acid (pH 6.0) and blocked with Bovine Serum Albumin (BSA). The sections were incubated with rabbit anti- HIF1α (1:200, Abcam, USA) or rabbit anti- phospho-CaMKII (1:200, CST, USA) at 4°C overnight. Then the sections were incubated with goat anti-rabbit IgG-HRP secondary antibody (1:200, Bioss, China) at room temperature for 60 min. Visualized staining of tissue was performed with a fluorescence microscope.

### Glucose uptake assays

The Cell Meter™ 2-NBDG [2-(N-(7-Nitrobenz-2-oxa-1,3-diazol-4-yl)amino)-2-deoxyglucose] Glucose Uptake Assay Kit (AAT Bioquest Inc., Sunnyvale, CA, USA) was used to monitor the glucose transporters. 2-NBDG staining solution was added to prepared BMDMs for 20 min at 37°C. After staining, the staining solution was removed, and cells were washed with an assay buffer. Cells were analyzed using a fluorescence microscope or flow cytometer.

### Single-cell RNA-seq analysis

ScRNA-seq data were downloaded from the Gene Expression Omnibus (GEO) database (GSE1555340). RNA-Seq Piezo1_KO (GSM4699498) and RNA-Seq Piezo1_WT (GSM4699499) were chosen to be analyzed (33). The R package Seurat was used for data analysis. Cells with > 5% mitochondrial genes or < 200 distinct genes were filtered. Data were normalized and scaled using the LogNormalize method (scale factor = 10,000). Variable genes were detected using FindVariable Features. Cells were clustered using the FindClusters method (resolution = 0.5) and visualized using t-distributed stochastic neighbor embedding (t-SNE). Differentially expressed genes (DEGs) were determined using the FindAllMarkers function. GO analysis was performed by enrichGO.

### DSS-induced colitis

Age- and sex-matched control and experimental mice were administered 3% DSS (Meilun Biotechnology Co., Ltd., China) in drinking water for 7 days. Body weight was monitored every 24 h. On day 7, mice were anesthetized and sacrificed and the length of the entire colon was measured. Colitis was assessed using hematoxylin and eosin staining, and severity scores were calculated. Histological evaluation of DSS-induced colitis. The evaluation of the histological changes in the colon was performed according to the published criteria (35). The scoring system was put in **Supplementary Table 2**.

### Isolation of the intestinal macrophages from lamina propria mononuclear cells

LPMCs were isolated as previously described (36). Briefly, colonic tissue was cut into 1-mm slices and the epithelium was eliminated by HBSS/EDTA for 30 min with stirring at 37°C (twice). Tissue pieces were washed with PBS and collected in RPMI containing collagenase IV (Worthington Industries, Columbus, OH, USA), DNase I (Sigma-Aldrich), and dispase II (Sigma-Aldrich) with gentle shaking at 37°C for 60 min. LPMCs were purified by centrifugation through a 35%/80% discontinuous Percoll gradient (Solarbio Life Science, Beijing, China).

### LC-MS analysis of metabolites

Metabolite quantification was conducted by Shanghai BIOTREE BIOTECH CO., LTD using liquid chromatography-tandem mass spectrometry (LC-MS/MS). The samples were vortexed for 1 min after adding 1000 μL of precooled MeOH/H_2_O (3/1, v/v). Samples were precooled in dry ice, freeze-thawed in liquid nitrogen, and vortexed for 30 s. Cells were then sonicated for 15 min in an ice-water bath, shaken (vibrated) at 4°C for 15 min, incubated at −40°C for 1 h, and then centrifuged at 12000 rpm for 15 min. An 800 μL aliquot of the clear supernatant was collected and dried by centrifugation. Dried samples were then reconstituted in 150 µL ultrapure water, vortexed prior to filtration through a centrifuge tube filter, and subsequently transferred to inserts in injection vials for HPIC-MS/MS analysis. Electrospray ionization mass spectrometry was performed using a SCIEX 6500 QTRAP+ triple quadrupole mass spectrometer in the negative mode. The final concentration (CF, nmol · L−1) was equal to the calculated concentration (CC, nmol · L−1) multiplied by the dilution factor (Dil).

### Calcium imaging

BMDMs were cultured with the indicated conditons. Thereafter, the cells were loaded with Fluo-4 AM (2.5 μM; TermoFisher, USA) for 15-30 min in phosphate-buffered saline (PBS) at 37°C to observe calcium influx. The intracellular calcium ions showed green fluorescence. Images were captured with a fluorescence microscope.

### Statistical analysis

All experiments were repeated at least thrice. Data are presented as mean ± standard deviation. Shapiro-Wilk test was used for normality tests. Comparisons between groups were performed using one-way ANOVA or unpaired two-tailed Student’s t-tests. Statistical significance was set at *P* < 0.05.

## Results

### Piezo1 activation promotes LPS-induced expression and secretion of inflammatory cytokines

**Figure 1:**
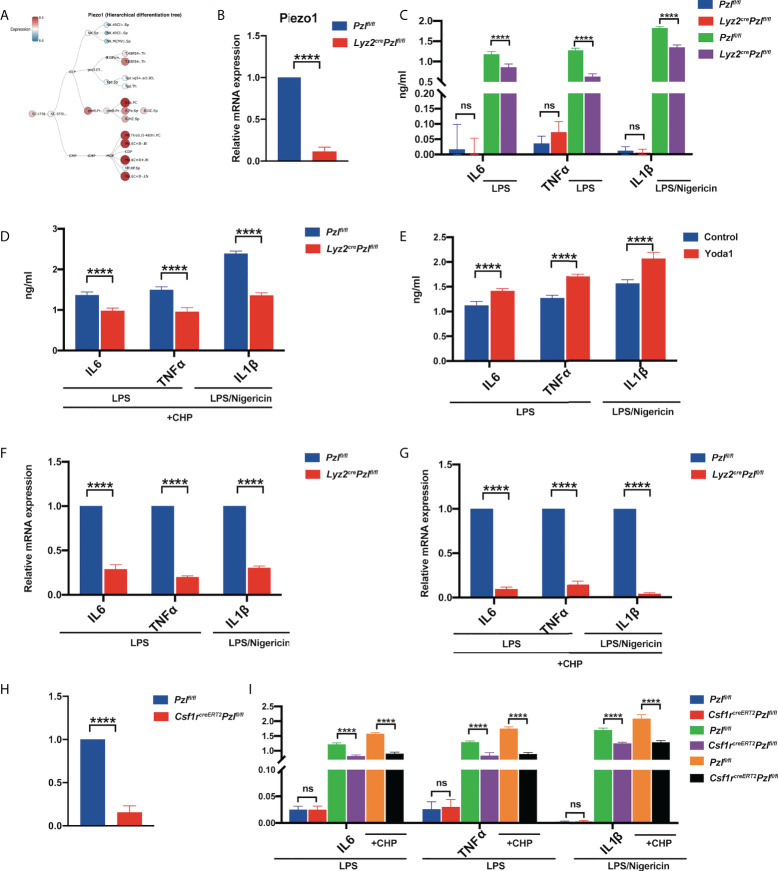
Piezo1 activation promotes LPS-induced expression and secretion of inflammatory cytokines. **(A)** A hierarchical differentiation tree depicting the expression of Piezo1 using BloodSpot (online software). **(B)** The expression of Piezo1 mRNA in the BMDMs from *Lyz2* ^cre/+^ *Piezo1* ^flox/flox^ and *Piezo1* ^flox/flox^ control mice relative to β-actin (n = 6). **(C)** IL6 and TNF-α levels in the BMDMs from *Lyz2* ^cre/+^ *Piezo1* ^flox/flox^ mice and *Piezo1* ^flox/flox^ control mice treated with 10 ng/ml LPS for 24 h; IL-1β level in the BMDMs from *Lyz2* ^cre/+^ *Piezo1* ^flox/flox^ mice and *Piezo1* ^flox/flox^ control mice treated with 1 µg/ml LPS for 4 h and 25 µM nigericin for 30 min, measured by ELISA (n = 6). **(D)** IL6 and TNF-α levels in the BMDMs from *Lyz2* ^cre/+^ *Piezo1* ^flox/flox^ mice and *Piezo1* ^flox/flox^ control mice treated with 10 ng/ml LPS under cyclical hydrostatic pressure cycling once per second from 45 mmHg to 60 mmHg with 5% CO_2_ at 37°C for 6 h; IL-1β level in the BMDMs from *Lyz2* ^cre/+^ *Piezo1* ^flox/flox^ mice and *Piezo1* ^flox/flox^ control mice treated with 1 µg/ml LPS for 4 h and 25 µM nigericin for 30 min, measured by ELISA under CHP (n = 6). **(E)** IL6 and TNF-α levels in the BMDMs with 10 ng/ml LPS for 24 h pretreated with 10 µM Yoda1 or DMSO for 30 min; IL-1β level in the BMDMs with 1 µg/ml LPS for 4 h and 25 µM nigericin for 30 min pretreated with 10 µM Yoda1 or DMSO for 30 min (n = 6). **(F)** Relative gene expression of IL6, TNF-α, and IL-1β in the BMDMs from *Lyz2* ^cre/+^ *Piezo1* ^flox/flox^ mice and *Piezo1* ^flox/flox^ control mice incubated with 10 ng/ml LPS for 2 h or 1 ug/ml LPS for 4 h and 25 µM nigericin for 30 min (n = 6). **(G)** Relative gene expression of IL6 and TNF-α in the BMDMs from *Lyz2* ^cre/+^ *Piezo1* ^flox/flox^ mice and *Piezo1* ^flox/flox^ control mice treated with 10 ng/ml LPS for 2 h under cyclical hydrostatic pressure; relative gene expression of IL-1β in the BMDMs treated with 1 ug/ml LPS for 4 h and 25 µM nigericin for 30 min (n = 6). **(H)** The expression of Piezo1 mRNA in BMDMs from *Csf1r* ^creERT2^; *Piezo1* ^flox/flox^ and *Piezo1* ^flox/flox^ control mice relative to β-actin (n = 6). **(I)** IL6, TNF-α, and IL-1β secretion in the BMDMs from *Csf1r* ^creERT2^; *Piezo1* ^flox/flox^ mice and *Piezo1* ^flox/flox^ control mice incubated with 10 ng/ml LPS for 24 h, 1 µg/ml LPS for 4 h, and 25 µM nigericin for 30 min under static pressure or cyclical hydrostatic pressure (n = 6). Statistical significances were calculated using **(C, I)** one-way ANOVA, Tukey’s multiple comparisons test; **(B, D–H)** two-tailed Student t test. Data are expressed as the mean ± SD. **** *P* < 0.0001, ns, not significant. BMDMs, bone marrow-derived macrophages; *Lyz2* ^cre/+^ *Piezo1* ^flox/flox^, conditionally Piezo1-deficient mice; IL, interleukin; TNF-α, tumor necrosis factor α; LPS, liposaccharide; CHP, cyclical hydrostatic pressure.

First, we investigated the Piezo1 mRNA expression profile in hematopoietic cells at various differentiation stages using the BloodSpot database. The hierarchical differentiation tree showed that Piezo1 was generally more highly expressed in myeloid cells than in lymphoid cells (**Figure 1A**). We examined the mRNA expression of several genes encoding mechanosensitive channels in BMDMs and found that Piezo1 was highly expressed in BMDMs under homeostasis, LPS treatment and CHP treatment (**Supplementary Figure 1A**).

To investigate how Piezo1 regulates macrophage function, *Lyz2* ^cre/+^ *Piezo1* ^flox/flox^ mice were generated with Piezo1 conditional knockout in myeloid cells. Sex-matched littermates (*Piezo1* ^flox/flox^) served as the controls. Piezo1 deletion in BMDMs was verified by RT-qPCR (**Figure 1B**). There were no differences in myeloid differentiation between these two types of mice, and the morphology of BMDMs was also comparable (**Supplementary Figures 1B–D**).

It was reported that TLR4 signaling augmented macrophage bactericidal activity through the mechanical sensor Piezo1 (34). To prove that Piezo1 deficiency could affect TLR4-mediated immune responses in BMDMs, we detected IL-6 and TNF-α secretion after LPS stimulation. In particular, the release of IL-1β requires NLRP3 inflammasome activation mediated by LPS (first signal, “priming”) and nigericin (second signal, “activation”) (37–41). Our results showed that Piezo1 deficiency reduced IL-6 and TNF-α production upon LPS stimulation and reduced IL-1β production in LPS/nigericin stimulated BMDMs after static pressure (**Figure 1C**) and CHP (**Figure 1D**) treatments. Moreover, pretreatment with the Piezo1 agonist, Yoda1, promoted LPS-or LPS/nigericin-induced secretion of inflammatory cytokines (**Figure 1E**) in BMDMs. Consistently, LPS-or LPS/nigericin -induced IL-6, TNF-α, and IL-1β transcription levels were also significantly reduced in Piezo1-deficient BMDMs after static pressure (**Figure 1F**) and cyclic hydrostatic pressure (**Figure 1G**) treatments. Additionally, almost all BMDMs harvested from *Csf1r-EGFP* reporter mice expressed EGFP, suggesting that Csf1r is a good marker to label macrophages (**Supplementary Figure 1E**). To further ensure accurate data interpretation and avoid the impact of Piezo1 on BMDM induction, we used another mouse line, tamoxifen-inducible Cre recombinase (creERT2) under the control of the *Csf1r* promoter, crossed with *Piezo1* ^flox/flox^ transgenic mice. Piezo1 was depleted in BMDMs of *Csf1r* ^creERT2^ *Piezo1* ^flox/flox^ transgenic mice after tamoxifen treatment, and Piezo1 depletion decreased the expression of LPS-or LPS/nigericin-induced inflammatory cytokines (**Figures 1H, I**).

### Piezo1 is essential for glycolysis reprogramming in macrophages

**Figure 2:**
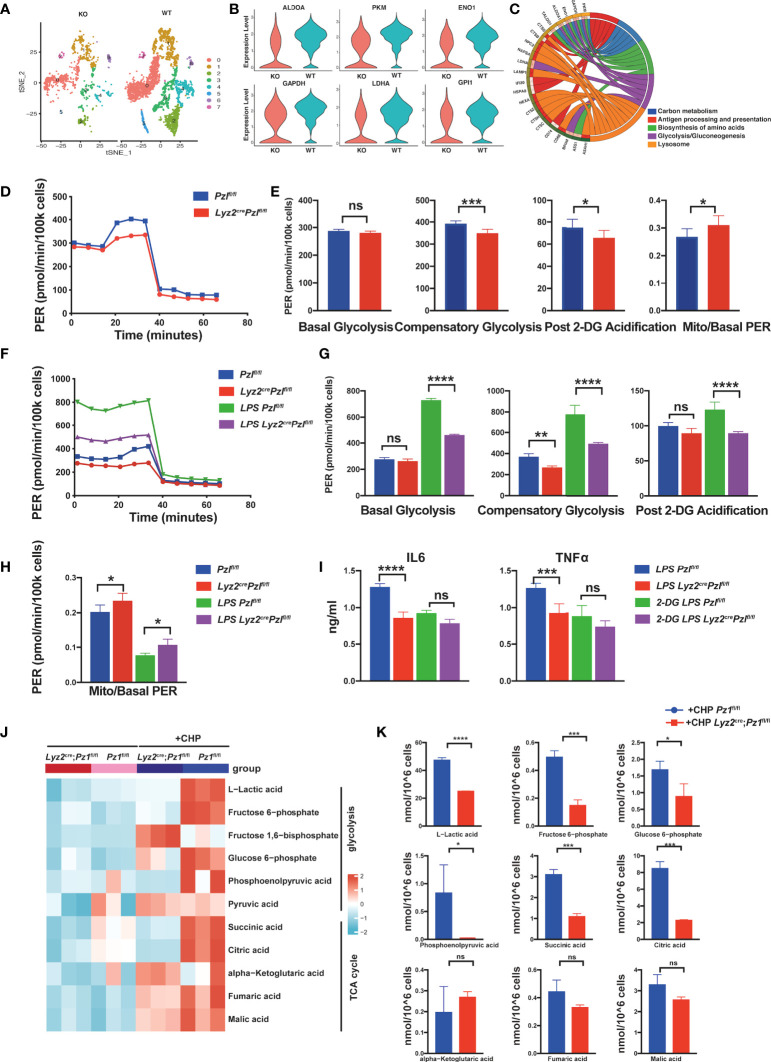
Piezo1 is essential for glycolic reprogramming in macrophages. **(A)** A t-SNE plot of myeloid gene clusters of myeloid cells harvested from the bone marrow of *Lyz2* ^cre/+^ *Piezo1* ^flox/flox^ mice and littermate controls, analyzed by scRNA-seq. **(B)** Violin plot showing expression levels of Aldoa, Pkm, Eno1, Gapdh, Ldha, and Gpi1. **(C)** GO analysis of the top five enriched pathways based on differentially expressed genes (DEGs). **(D, E)** Glycolytic rate assay profile and glycolytic analysis of BMDMs from *Lyz2* ^cre/+^ *Piezo1* ^flox/flox^ mice and *Piezo1* ^flox/flox^ control mice (n = 7). **(F–H)** Glycolytic rate assay profile and glycolytic analysis of BMDMs from *Lyz2* ^cre/+^ *Piezo1* ^flox/flox^ mice and *Piezo1* ^flox/flox^ control mice treated with or without LPS (10 ng/ml) for 24 h (n = 7). **(I)** IL6 and TNF-α levels in BMDMs from *Lyz2* ^cre/+^ *Piezo1* ^flox/flox^ mice and *Piezo1* ^flox/flox^ control mice treated with or without LPS (10 ng/ml) and 2-DG (1 mM) for 24 h (n = 6). **(J, K)** Quantitative proteomics was performed to assess metabolites related to glucose metabolism in the BMDMs from *Lyz2* ^cre/+^ *Piezo1* ^flox/flox^ mice and *Piezo1* ^flox/flox^ control mice under static pressure or cyclical hydrostatic pressure (n = 3). Statistical significances were calculated using **(G–I)** one-way ANOVA, Tukey’s multiple comparisons test; **(E, K)** two-tailed Student t test. Data are expressed as mean ± SD. *P < 0.05, **P < 0.01, ***P < 0.001; **** *P* < 0.0001, ns, not significant. BMDMs, bone marrow-derived macrophages; *Lyz2* ^cre/+^ *Piezo1* ^flox/flox^, conditionally Piezo1-deficient mice; IL, interleukin; TNF-α, tumor necrosis factor α; LPS, liposaccharide.

To determine the mechanisms of the proinflammatory profile induced by Piezo1 deficiency, we analyzed published scRNA-seq data (GSE155340) of myeloid cells harvested from the bone marrow of *Lyz2* ^Cre/+^ *Piezo1* ^fl/fl^ mice and littermate control mice (33). The results revealed that the downregulated genes in the Piezo1-deficient cells were significantly involved in the biological processes of carbon metabolism in GO analysis, suggesting the role of Piezo1 in glucose metabolism. Additionally, the expression of glycolysis-related genes (Alodoa, Pkm, Eno1, Gapdh, Ldha, and Gpi1) was decreased in Piezo1-deficient myeloid cells (**Figures 2A–C**).

Next, we performed an XF glycolytic rate assay to validate glycolytic changes in Piezo1-deficient macrophages. Piezo1 deficiency decreased compensatory glycolysis and post 2-DG (2-deoxyglucose) acidification both in BMDMs and peritoneal macrophages, even without LPS stimulation. However, the mitoOCR (oxygen consumption rate)/basal PER (proton efflux rate) ratio was increased in Piezo1-depleted macrophages, which showed a tendency towards OXPHOS when Piezo1 was completely depleted (**Figures 2D, E** and **Supplementary Figures 2A, B**). The glycolytic rate in *Csf1r* ^creERT2^ *Piezo1* ^flox/flox^ macrophages supported the conclusion that Piezo1 contributed to the glycolytic reaction (**Supplementary Figures 2C–F**).

Furthermore, we conducted a glycolytic rate analysis of LPS-treated BMDMs to investigate the effect of Piezo1 on LPS-induced metabolic remodeling. As expected, LPS stimulation significantly increased the rate of glycolysis in BMDMs. Basal glycolysis, compensatory glycolysis, and post 2-DG acidification of LPS-stimulated Piezo1-depleted BMDMs were distinctly lower than those of LPS-stimulated control BMDMs (**Figure 2F, G**), whereas the mitoOCR/basal PER ratio was higher in LPS-stimulated Piezo1-depleted BMDMs (**Figure 2H**). This result indicated that Piezo1 deficiency disrupted LPS-induced metabolic reprogramming. In addition, we used a fluorescently tagged glucose tracer, 2-NBDG, to monitor glucose transportation in BMDMs, and flow cytometry revealed that Piezo1 deficiency restrained glucose uptake upon LPS stimulation (**Supplementary Figure 2G**). Importantly, the d-glucose mimic, 2-DG, inhibited LPS-induced IL-6 and TNF-α secretions in Piezo1-depleted BMDMs, suggesting that Piezo1-mediated metabolic reprogramming affects the immune response induced by macrophages (**Figure 2I**).

Since Piezo1 is activated by external mechanical cues, we used metabolomics to further analyze the changes in the key products of glucose metabolism in BMDMs with or without CHP treatment. CHP prominently increased multiple intermediate products of glycolysis, indicating enhanced glucose metabolism (**Figure 2J**). Upon CHP stimulation, several products of glycolysis, especially lactic acid, which is the end-product of glycolysis, were significantly reduced in Piezo1-deficient BMDMs compared to wild-type BMDMs. This result validated the reduced glycolysis in Piezo1-deficient BMDMs. In contrast, TCA cycle metabolites showed varying degrees of accumulation. Succinic acid and citric acid exhibited higher accumulation levels in wild-type BMDMs after CHP treatment compared to those in Piezo1-deficient BMDMs, whereas fumaric acid and malic acid were comparable (with no statistically significant differences) between these two groups, suggesting that Piezo1 is necessary for the CHP-induced truncated TCA cycle (**Figure 2K**).

### Yoda1 induces glycolysis in macrophages

**Figure 3:**
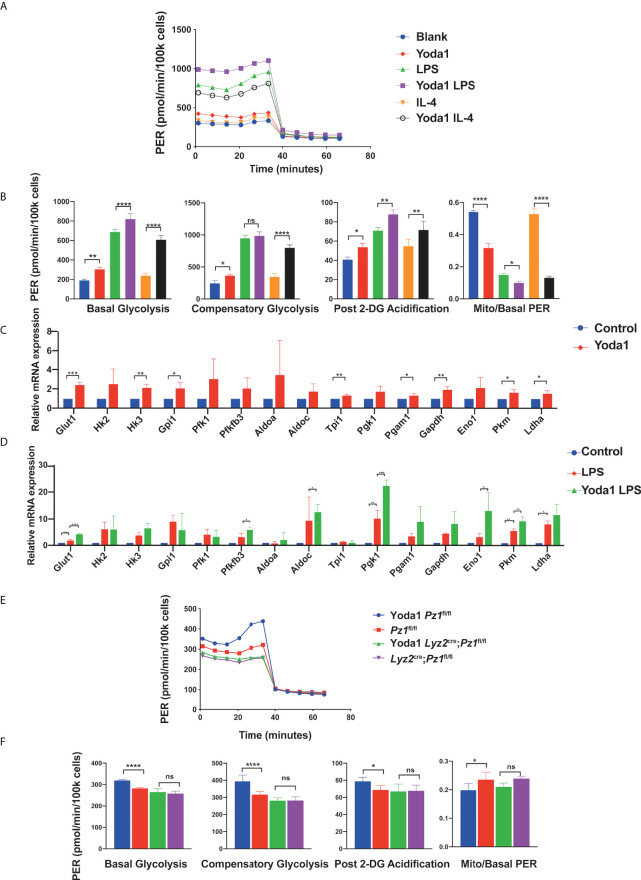
Yoda1 induces glycolysis in macrophages. **(A, B)** Glycolytic rate assay profile and glycolytic analysis of BMDMs pretreated with 10 µM Yoda1 or DMSO, incubated in the indicated conditions (blank; 10 ng/ml LPS for 24 h; 10 ng/ml IL4 for 24 h) (n = 4) **(C)** The mRNA expression of glycolysis-related genes treated with 10 µM Yoda1 or DMSO for 30 min (n = 3). **(D)** The mRNA expression of glycolysis-related genes pretreated with 10 µM Yoda1 or DMSO for 30 min and 10 ng/ml LPS for 2 h (n = 3). **(E, F)** Glycolytic rate assay profile and glycolytic analysis of the BMDMs from *Lyz2* ^cre/+^ *Piezo1* ^flox/flox^ mice and *Piezo1* ^flox/flox^ control mice pretreated with or without 10 µM Yoda1 for 30min (n = 7). Statistical significances were calculated using **(B, D, F)** one-way ANOVA, Tukey’s multiple comparisons test; **(C)** two-tailed Student t test. Data expressed as means ± SD. *P < 0.05, **P < 0.01, ***P < 0.001; **** P < 0.0001, ns, not significant. BMDMs, bone marrow-derived macrophages; *Lyz2* ^cre/+^ *Piezo1* ^flox/flox^, conditionally Piezo1-deficient mice; IL, interleukin; TNF-α, tumor necrosis factor α; LPS, liposaccharide.

Yoda1 is a specific Piezo1 channel agonist that stabilizes the open conformation of the channel and reduces its mechanical activation threshold (42, 43). It activates the Piezo1 channel in the absence of mechanical stimuli (44). To determine whether Yoda1 participates in the regulation of glucose metabolism, harvested BMDMs pretreated with or without Yoda1 were assayed using a Seahorse extracellular flux analyzer. Yoda1 preconditioning increased the glycolytic flux in BMDMs (**Figures 3A, B**). Yoda1 increased basal glycolysis, compensatory glycolysis, and post 2-DG acidification in unstimulated cells but decreased the mitoOCR/basal PER ratio. Additionally, LPS stimulation significantly increased the glycolysis capacity, and Yoda1 further enhanced this effect. Surprisingly, the increase in compensatory glycolysis in LPS-sensitized BMDMs did not show a significant difference after Yoda1 pretreatment, which could be due to the detection threshold. In addition, the mitoOCR/basal PER ratio in BMDMs markedly increased upon IL-4 stimulation, reflecting a metabolic conversion towards OXPHOS in alternatively activated macrophages, which was reversed by Yoda1 treatment. Yoda1 treatment promoted the expression of multiple glycolysis-related genes in both unstimulated (**Figure 3C**) and LPS-stimulated BMDMs (**Figure 3D**). To validate that Yoda1-induced metabolic regulation is dependent on the Piezo1 ion channel, we investigated the glycolytic rate of normal or Piezo1-deficient BMDMs pretreated with Yoda1 (**Figures 3E, F**). As expected, Piezo1 depletion reduced the promoting effect of Yoda1 on glycolysis in BMDMs, indicating that Yoda1-mediated metabolic regulation is Piezo1 dependent.

### Piezo1 promotes the metabolic switch in macrophages by regulating the Ca2+-CaMKII-HIF1α axis

**Figure 4:**
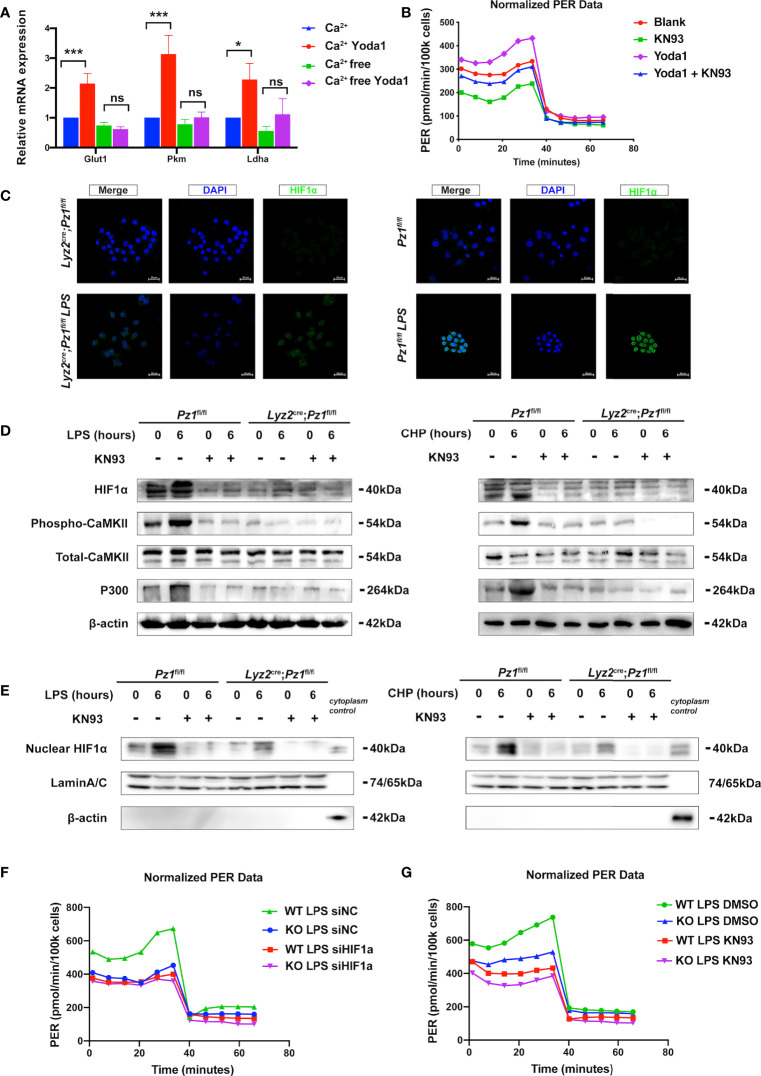
Piezo1 promotes a metabolic switch in macrophages *via* the Ca^2+^-CaMKII-HIF1α axis. **(A)**The mRNA expression of Glut1, Pkm, and Ldha treated with 10 µM Yoda1 or DMSO for 30 min in calcium-rich or calcium-free media. (n=3, one-way ANOVA, Tukey’s multiple comparisons test) **(B)** Glycolytic rate assay profile of BMDMs treated with blank (DMSO); KN93 (10uM KN93 for 24 h); Yoda1 (10uM Yoda1 for 30 min and overnight culture); Yoda1+KN93 (10uM Yoda1 for 30 min and 10uM KN93 for 24 h) **(C)** Immunofluorescence of HIF1α in the BMDMs from *Lyz2* ^cre/+^ *Piezo1* ^flox/flox^ mouse and *Piezo1* ^flox/flox^ control mice treated with or without LPS (10 ng/ml) for 24 h. **(D, E)** HIF1α, phospho-CaMKII, total-CaMKII, P300, β-actin, and LaminA/C, nuclear HIF1α expression in BMDMs in the indicated conditions (10 ng/ml LPS for 0 or 6 h, 10 µM KN93 for 0 or 6 h, static pressure or cyclical hydrostatic pressure for 0 or 6 h). **(F)** Glycolytic rate assay profile of BMDMs from *Lyz2* ^cre/+^ *Piezo1* ^flox/flox^ mice or *Piezo1* ^flox/flox^ control mice treated with siNC or siHIF1α. **(G)** Glycolytic rate assay profile of BMDMs from *Lyz2* ^cre/+^ *Piezo1* ^flox/flox^ mice or *Piezo1* ^flox/flox^ control mice treated with DMSO or 10 uM KN93 for 24 h. Data are expressed as the mean ± SD. *P < 0.05, ***P < 0.001; ns, not significant. BMDMs, bone marrow-derived macrophages; *Lyz2* ^cre/+^ *Piezo1* ^flox/flox^, conditionally Piezo1-deficient mice; HIF1α, hypoxia-inducible factor 1α.

Mechanistically, Piezo1 mediates extracellular Ca^2+^ influx and intracellular Ca^2+^ overload, thereby increasing the activation of calcium-dependent signaling (45, 46). Therefore, we used a calcium-free medium to deplete extracellular calcium ions to evaluate the transcription of glycolysis-related genes in Yoda1-treated BMDMs. First, Fluo-4 AM assay showed that Yoda1 promoted Calcium influx into the cytosol, and calcium elimination depletion decreased the calcium influx induced by Yoda1 (**Supplementary Figures 3A**). Calcium elimination depletion diminished the effects of Yoda1 on undifferentiated Glut1, Pkm, and Ldha mRNA levels (**Figure 4A**). Furthermore, KN93 reduced Yoda1-induced increase of glycolytic level (**Figure 4B**, **Supplementary Figures 3B**). Meanwhile, KN93 also reduced mRNA levels of glycolytic enzymes induced by Yoda1 (**Supplementary Figures 3C**). HIF1α is a primary transcription factor that regulates the expression of multiple glycolysis-associated genes; therefore, we performed immunofluorescence analysis of HIF1α, which revealed that Piezo1 knockout decreased the LPS-induced HIF1α expression and nuclear accumulation (**Figure 4C**). In addition, we used western blotting to determine the relative protein levels in Piezo1-deficient BMDMs, which revealed that the protein levels of HIF1α and its co-activator P300 were reduced in Piezo1-deficient macrophages (**Figure 4D**). The nuclear HIF1α protein level was also reduced (**Figure 4E**).

CaMKII activation, which can be induced by Piezo1 activation, has been reported to be involved in HIF1α stabilization (47, 48). We found that LPS stimulation or CHP markedly promoted CaMKII phosphorylation in normal cells whereas Piezo1 knockout reduced CaMKII phosphorylation by disturbing calcium homeostasis. Moreover, KN93 is a cell-permeable, reversible, and competitive inhibitor of CaMKII enzymatic activity. The administration of KN93 inhibited the phosphorylation of CaMKII and the LPS- or CHP-induced HIF1α stabilization and P300 expression in wild-type macrophages compared to Piezo1-deficient cells (**Figure 4D**). Then we used siHif1α to block the translation of Hif1α in BMDMs to observe the influence of Hif1α on glycolysis change induced by Piezo1 deficiency. Seahorse metabolic assay showed that downregulation of Hif1α eliminated the difference between normal BMDMs and Piezo1-deficient BMDMs (**Figure 4F**, **Supplementary Figures 3E**). Similarly, KN93 also attenuated the metabolic difference caused by Piezo1 knockout (**Figure 4G**, **Supplementary Figures 3F**). Collectively, these results indicate that Ca^2+^-CaMKII-HIF1α signaling is involved in Piezo1-induced metabolic reprogramming.

### Piezo1 deficiency ameliorates murine ulcerative colitis

**Figure 5:**
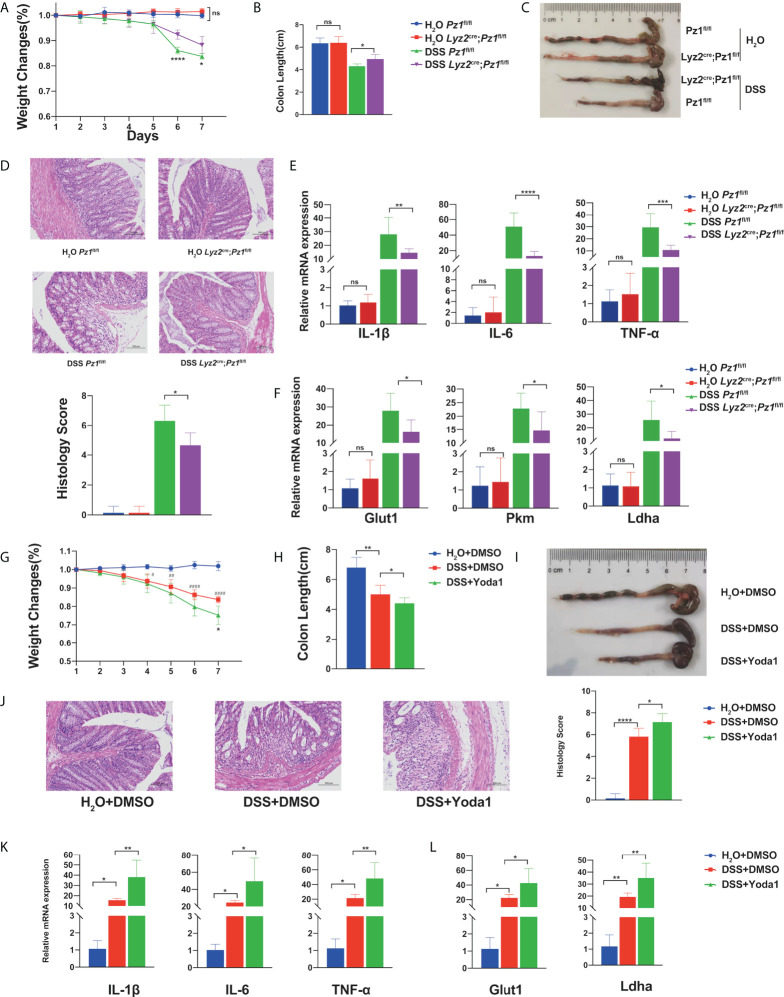
Piezo1 deficiency ameliorates murine ulcerative colitis*. Lyz2* ^cre/+^ *Piezo1* ^flox/flox^ and *Piezo1* ^flox/flox^ control mice were challenged with water or 3% DSS in drinking water for seven days (n = 7). **(A)** Body weight loss, **(B, C)** colon length, **(D)** representative hematoxylin and eosin **(H&E)**-stained colon cross-sections, and histological scores were measured. **(E)** mRNA expression of IL-1β, IL6, and TNF-α in colon tissue. **(F)** mRNA expression of Glut1, Pkm, and Ldha in macrophages in the lamina propria. Mice were challenged with 3% DSS in drinking water for 7 d, to which 0.4 mg/kg Yoda1 or DMSO were added on day 1 and day 4 (n = 5). **(G)** Body weight loss, **(H, I)** colon length, **(J)** representative H&E-stained colon cross-sections, and histological scores were measured. **(K)** mRNA expression of IL-1β, IL6, and TNF-α in the colon tissue. **(L)** mRNA expression of Glut1, Pkm, and Ldha in macrophages of the lamina propria. Statistical significances were calculated using **(A)** two way ANOVA, *P < 0.05; **** P < 0.0001 (DSS *Piezo1* ^flox/flox^ versus DSS *Lyz2* ^cre/+^ *Piezo1* ^flox/flox^); **(G)** two way ANOVA, *P < 0.05 (DSS+DMSO versus DSS+Yoda1); ^#^P < 0.05, ^# #^P < 0.01, ^####^P < 0.0001 (H_2_O+DMSO; DSS+DMSO); **(B–F, H–L)** one-way ANOVA, Tukey’s multiple comparisons test. Data are expressed as the mean ± SD. *P < 0.05, **P < 0.01, ***P < 0.001; **** P < 0.0001, ns, not significant. *Lyz2* ^cre/+^ *Piezo1* ^flox/flox^, conditionally Piezo1-deficient mice; DSS, dextran sulfate sodium; IL, interleukin; TNF-α, tumor necrosis factor α.

Intestinal inflammation is accompanied by changes in mechanical forces, including the increase of intraluminal pressures, colonic blood flow and matrix stiffness (49–54). DSS-induced murine ulcerative colitis is a self-limiting disease characterized by acute inflammation in the colon which is highly regulated by the macrophage-initiated innate immune response (55). Therefore, we aimed to confirm the role of Piezo1 *in vivo* in a DSS-induced colitis mouse model using transgenic mice. No significant differences in body weight, colon length, histological score, or inflammatory cytokines were observed between the *Lyz2* ^Cre/+^ *Piezo1* ^fl/fl^ and littermate control mice in the absence of inflammation. However, Piezo1 deficiency markedly ameliorated DSS-induced inflammation, with higher body weights (**Figure 5A**), longer colon length (**Figures 5B, C**), decreased histological score (**Figure 5D**), and lower levels of inflammatory cytokines (**Figure 5E**). In addition, the published scRNA-seq data (GSE138902) showed that Piezo1 was expressed in a substantial part of recruited macrophages during colitis. (**Supplementary Figures 4A**), while immunofluorescence results showed more macrophages recruitment in wild type mice than Piezo1-deficient mice (**Supplementary Figures 4B**). Consistent with our previous results, the mRNA levels of glycolysis-related genes in the Piezo1-deficient colonic macrophages were reduced (**Figure 5F**). Immunohistochemical figures also showed decreased HIF1α and phospho-CaMKII in colons of *Lyz2* ^cre/+^ *Piezo1* ^flox/flox^ compared with *Piezo1* ^flox/flox^ control mice in the DSS-induced colitis (**Supplementary Figures 4C**).

To determine whether Yoda1-induced Piezo1 activation is influenced by the progression of murine colitis, DSS-treated *Csf1r-EGFP* transgenic mice were intraperitoneally injected with Yoda1. The control group was injected with equal amounts of DMSO. First, we demonstrated that only Yoda1 treatment did not affect body weight, colon length and histological score compared with control group under physiological conditions (**Supplementary Figures 4D**). While, Yoda1 exacerbated disease progression compared to DMSO treatment upon DSS treatment. Loss of body weight (**Figure 5G**) and longer colon length (**Figures 5H, I**) were observed in the DSS+Yoda1 group compared to the DSS+DMSO group. Histological staining and immunofluorescence revealed that Yoda1 promoted immune cell infiltration (**Figure 5J** and **Supplementary Figure 4D**), as Piezo1 could affect LPS induced expression of chemokine Cxcl1 and Cxcl2 in BMDMs cultured *in vitro* (**Supplementary Figure 4E**). Yoda1 also resulted in increased IL-6, TNF-α, and IL-1β levels (**Figure 5K**), as well as increased Glut1 and Ldha mRNA levels in colonic macrophages (**Figure 5L**).

## Discussion

Mechanical forces are a main environmental feature of acute and chronic inflammation and may shape immune responses mediated by macrophages. In this study, we revealed that Piezo1 deficiency impaired LPS-induced cytokine secretion in macrophages, whereas Yoda1 or CHP treatment enhanced cytokine secretion. Mechanistically, Piezo1 deficiency attenuated aerobic glycolysis in resting or LPS-stimulated macrophages, whereas Yoda1 or CHP treatment upregulated the glycolytic activity. We further revealed that Piezo1 may regulate glycolytic activity in macrophages *via* the Ca^2+^-induced CaMKII-HIF1α axis. Moreover, Piezo1 deficiency ameliorated DSS-induced colitis by limiting the glycolytic level and cytokine secretion of macrophages *in vivo*, whereas Yoda1 treatment aggravated DSS-induced colitis. These findings indicate that Piezo1-mediated mechanosensation induces a metabolic switch towards aerobic glycolysis, which could potentiate the inflammatory response of macrophages. Our results provide insights into the mechanical stimulation-induced metabolic regulation in macrophages.

TLRs are critical pattern recognition receptors in macrophages which should be able to integrate other signals to distinguish different severity of invading pathogens to generate a proper immune response, avoiding insufficient or excessive inflammation (56). Several studies have suggested that increased intracellular Ca^2+^ plays an important role in mediating the TLR-triggered immune response (57, 58). Stromal interaction molecule 1 (STIM1) operated Ca^2+^ channels mediated Ca^2+^ influx can coordinate the activation of the small GTPases upon TLR stimulation, which enhance the activation of macrophages through a feedforward mechanism (59). The extracellular matrix (ECM) stiffness and architecture is one of the hallmarks of inflammation (60). A recent study has reported that LPS stimulation triggers assembly of the complex of Piezo1 and TLR4, and induces Piezo1-mediated Ca^2+^ influx, which demonstrated a direct connection between mechanosensation signal and TLR signal (34). So, we used LPS stimulation to induce Piezo1-mediated calcium influx and observe immune response of macrophages. On the other hand, classical studies first implicated ion channels to be mechanically activated (22), and it has been reported that CHP stimulated innate immune cells to mount an inflammatory response (31). Therefore, we chose CHP to serve as the mechanical stimulus for Piezo1 and demonstrate the potential role for Piezo1 in mechanosensation of macrophages. The variety of factors affecting the activation of Piezo1 suggests it is widely involved in multiple cellular biological processes, which deserves further investigation.

Ca^2+^ signals are crucial for controlling cellular energy metabolism, especially glycolysis, to maximize cellular glucose utilization during certain physiological or pathological processes (61). Purinergic receptor-mediated Ca^2+^ signals promote glucose uptake and aerobic glycolysis in astrocytes, with a robust increase in the concentrations of intracellular glucose and lactate (62). Mechanistically, several studies have emphasized the crucial role of HIF1α in Ca^2+^-induced glycolytic metabolism, as HIF1α translocates into the nucleus and initiates the transcription of multiple glycolytic genes (63, 64). In addition, HIF1α is stabilized under CHP and facilitates a prolonged proinflammatory expression profile in myeloid cells (31). Consistent with these studies, our data showed that Piezo1 activation upregulates glycolytic gene expression *via* the Ca^2+^-CaMKII-HIF1α axis, which plays a central role in the metabolic priming effect of Piezo1 activation on TLR signaling.

The cells in the intestinal tract experience various mechanical forces during peristalsis including compression, shear stress, strain, deformation and other forces. During intestinal inflammation, the increase of intraluminal pressures causes abnormal mechanical forces, which can have the reverse effect on gut physiology (49–51). Furthermore, substantial increase in colonic blood flow in IBD could increase capillary pressures and interstitial pressures (65). Under physiological conditions, the flexible gut is characterized by a rhythmic contractile pattern. While in the inflammatory diseases, the gut becomes rigid and fibrotic, and the mechanical properties of the intestinal wall correlate with severity of inflammation (52–54). Macrophages play pivotal roles in the immune response to harmful substances that breach the epithelial barrier in intestinal immune cells (66, 67). During pathological conditions such as inflammatory bowel disease (IBD), monocytes infiltrate the intestine in large numbers and tend to acquire a classically activated phenotype with a high secretion of inflammatory cytokines (68). Based on the findings, we revealed that Piezo1 activation is crucial for metabolic changes and inflammatory responses of macrophages in IBD, suggesting that the intestinal environment in IBD plays a role in the inflammatory function and phenotype of intestinal macrophages.

## Data availability statement

The original contributions presented in the study are included in the article/**Supplementary Material**. Further inquiries can be directed to the corresponding authors.

## Ethics statement

The animal study was reviewed and approved by the Animal Ethics Committee of Cheeloo College of Medicine, Shandong University.

## Author contributions

JP and XH designed the research, analyzed the data, and wrote the paper. SL performed the research, analyzed the data, and wrote the paper. XZ, SW, QL, AL, ZS, and QF performed the research and evaluated the data. All authors read and edited the manuscript. All authors contributed to the article and approved the submitted version.

## Funding

This work was supported by grants from the National Natural Science Foundation of China (No. 82030005, 91942306, 82170124, 81900122, 82000126), Rongxiang Regenerative Medicine Foundation of Shandong University (No. 2019SDRX-02) and China Postdoctoral Science Foundation (2020M672075).

## Acknowledgments

We thank Translational Medicine Core Facility of Shandong University for consultation and instrument availability that supported this work. And we would like to thank Editage (www.editage.com) for English language editing.

## Conflict of interest

The authors declare that the research was conducted in the absence of any commercial or financial relationships that could be construed as a potential conflict of interest.

## Publisher’s note

All claims expressed in this article are solely those of the authors and do not necessarily represent those of their affiliated organizations, or those of the publisher, the editors and the reviewers. Any product that may be evaluated in this article, or claim that may be made by its manufacturer, is not guaranteed or endorsed by the publisher.
