# Supplementary material for: Ion channel Piezo1 activation promotes aerobic glycolysis in macrophages
Source: Front Immunol. 2022 Sep 2;13:976482. doi: 10.3389/fimmu.2022.976482 (PMC9479104; doi:10.3389/fimmu.2022.976482)
Supplement: Supplementary file 1 [file DataSheet_1.docx]

Ion Channel Piezo1 Activation Promotes Aerobic Glycolysis in Macrophages

Supplementary Material

# Supplementary Figures


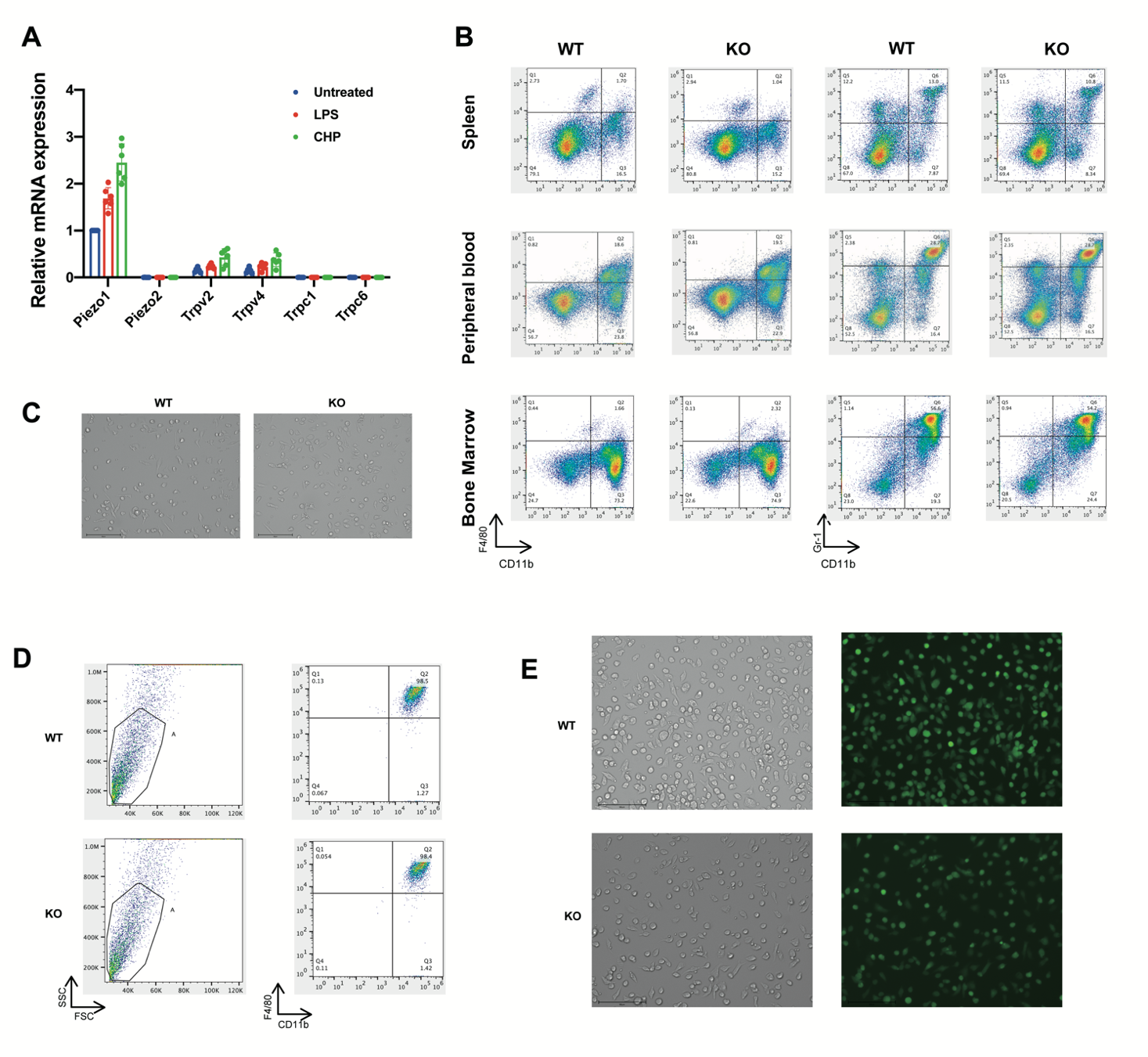


**Supplementary Figure 1.** (**A**) mRNA expression of several mechanosensitive channels in untreated BMDMs, BMDMs treated with 10 ng/ml LPS for 2 h, BMDMs treated with CHP for 2h (n=6). (**B-D**) Myeloid differentiation analysis and morphology of *Lyz2*^cre/+^*Piezo1*^flox/flox^ mice and *Piezo1*^flox/flox^ control mice. (**E**) Morphology and GFP expression of BMDMs from *Csf1r-EGFP* mice. Data expressed as means ± SD. **P* < 0.05, ***P* < 0.01, ****P* < 0.001; **** *P* < 0.0001, ns = not significant. BMDMs, bone marrow-derived macrophages; *Lyz2*^cre/+^*Piezo1*^flox/flox^ , conditionally Piezo1-deficient mice.


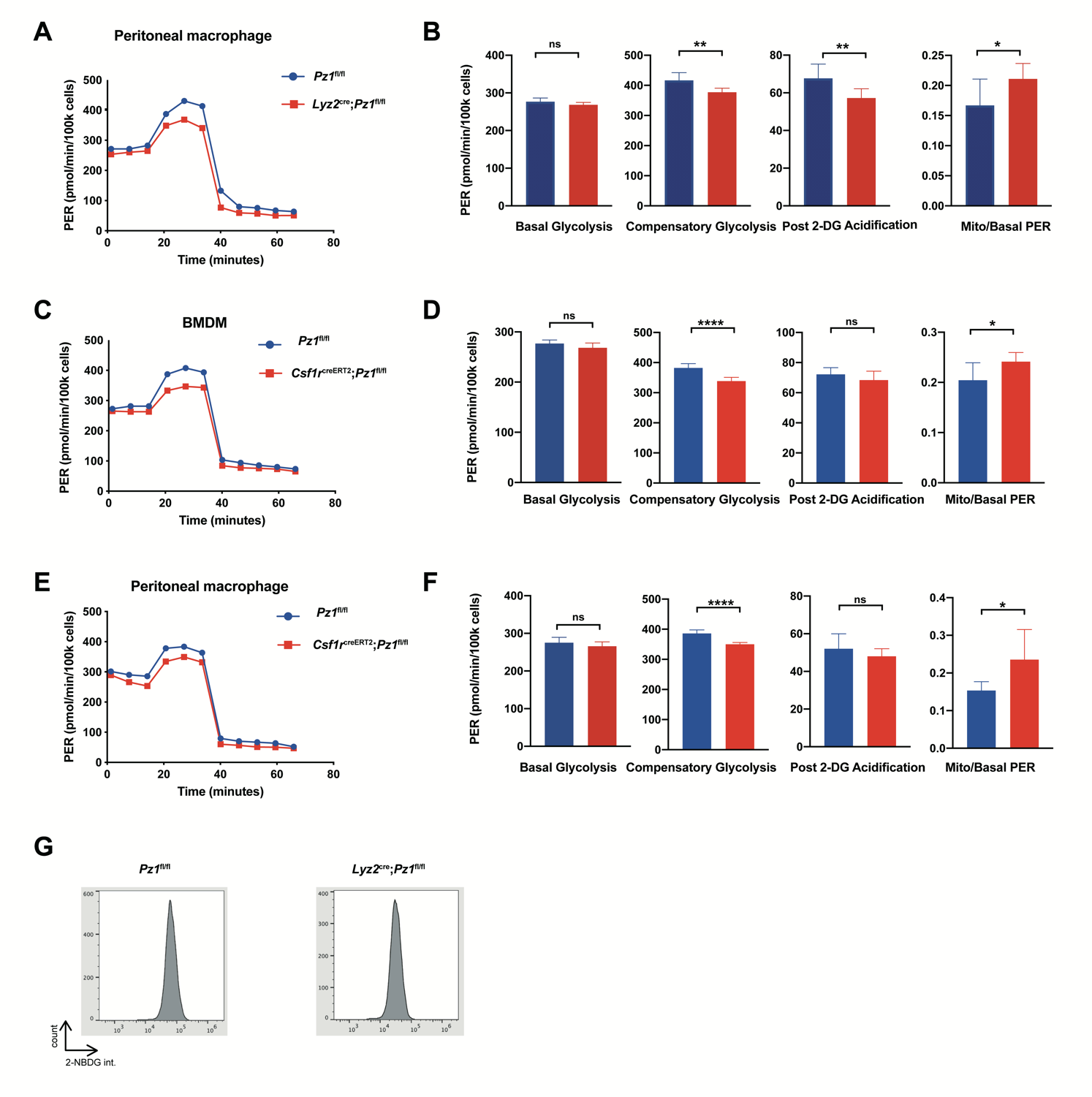


**Supplementary Figure 2.** (**A-B**) Glycolytic rate assay profile and glycolytic analysis of peritoneal macrophages of *Lyz2*^cre/+^*Piezo1*^flox/flox^ mice and *Piezo1*^flox/flox^ control mice (n=8). (**C-D**) Glycolytic rate assay profile and glycolytic analysis of BMDMs from *Csfr*^creERT2^, *Piezo1*^flox/flox^ mice and *Piezo1*^flox/flox^ control mice (n=7). **(E-F**) Glycolytic rate assay profile and glycolytic analysis of peritoneal macrophages of *Csf1r*^creERT2^, *Piezo1*^flox/flox^ mice and *Piezo1*^flox/flox^ control mice (n=8). (G)2-NBDG flow cytometry of BMDMs from *Lyz2*^cre/+^*Piezo1*^flox/flox^ mice and *Piezo1*^flox/flox^ control mice. BMDMs were pretreated with 10 ng/ml LPS for 6 hours. Statistical significances were calculated using two-tailed Student t test. Data are expressed as mean ± SD. **P* < 0.05, ***P* < 0.01, ****P* < 0.001; **** *P* < 0.0001, ns = not significant. BMDMs, bone marrow-derived macrophages; *Lyz2*^cre/+^*Piezo1*^flox/flox^, conditionally Piezo1-deficient mice.


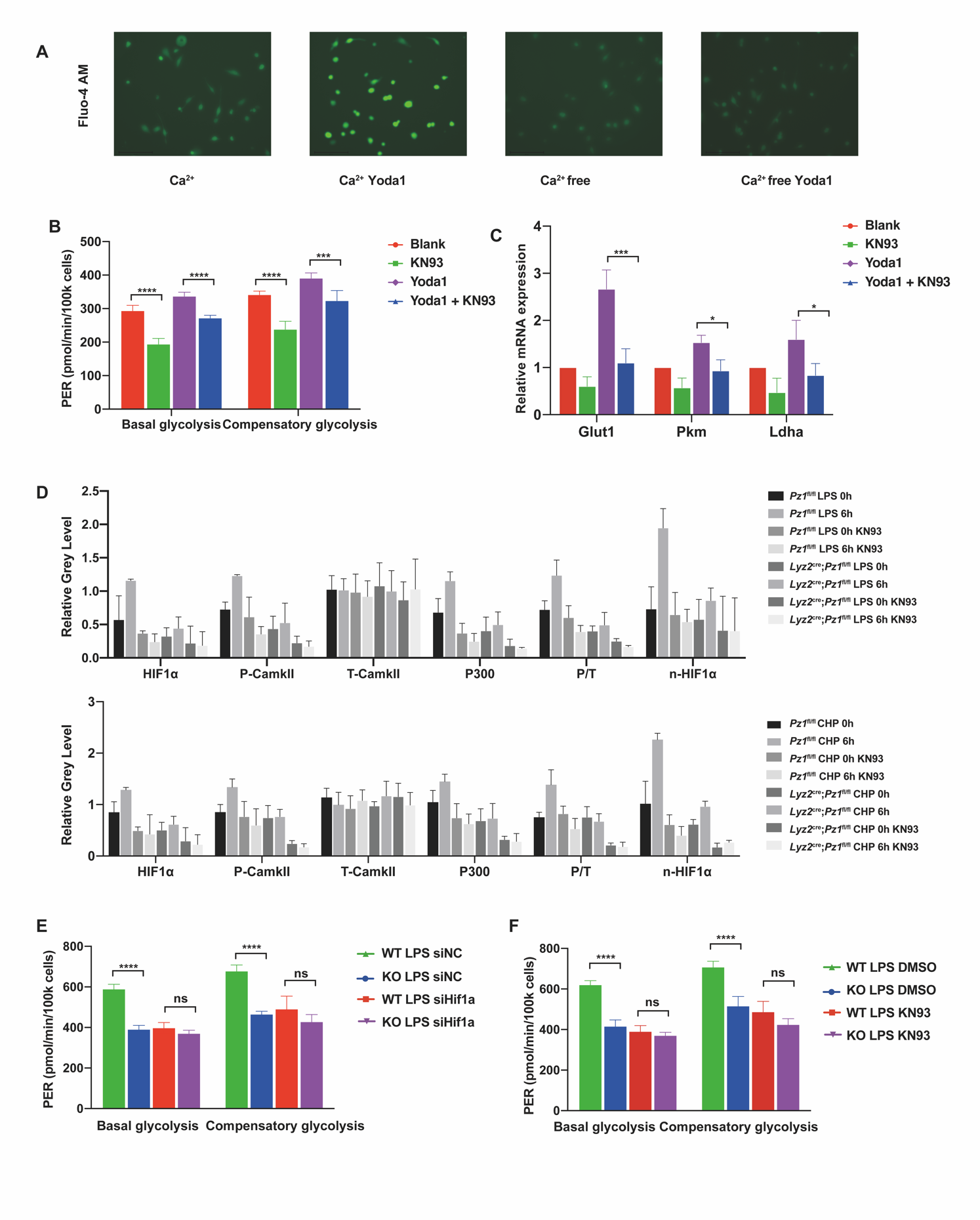


**Supplementary Figure 3.** (**A**) Calcium influx of BMDMs pretreated with Yoda1 or DMSO was tested by Fluo-4 AM. (**B**) Glycolytic rate assay analysis of BMDMs treated with blank (DMSO for 24 h); KN93 (10uM KN93 for 24 h); Yoda1 (10uM Yoda1 for 30 min and overnight culture); Yoda1+KN93 (10uM Yoda1 for 30 min and 10uM KN93 for 24 h) (n=5). (**C**) The mRNA expression of glycolysis-related genes of BMDMs treated with blank (DMSO for 2.5 h); KN93 (DMSO for 30 min and 10uM KN93 for 2 h); Yoda1 (10uM Yoda1 for 30 min and then DMSO for 2 h); Yoda1+KN93 (10uM Yoda1 for 30 min and 10uM KN93 for 2 h) (n=3). (D) Quantification of WB analysis of total HIF1α, phospho-CaMKII (P-CaMKII), total-CaMKII (T-CaMKII), P300, phospho-CaMKII / total-CaMKII (P/T ratio) relative to beta-actin; nuclear HIF1α (n-HIF1α) relative to LaminA/C in BMDMs in the indicated conditions (10 ng/ml LPS for 0 or 6 h, 10 µM KN93 for 0 or 6 h, static pressure or cyclical hydrostatic pressure for 0 or 6 h) (n = 3 for each group). (E) Glycolytic rate assay analysis of BMDMs from *Lyz2*^cre/+^*Piezo1*^flox/flox^ mice or *Piezo1*^flox/flox^ mice treated with siNC or siHIF1α (n=6). (F) Glycolytic rate assay analysis of BMDMs from *Lyz2*^cre/+^*Piezo1*^flox/flox^ mice or *Piezo1*^flox/flox^ control mice treated with DMSO or 10 uM KN93 for 24 h (n=6). Statistical significances were calculated using one-way ANOVA, Tukey’s multiple comparisons test. Data are expressed as mean ± SD. **P* < 0.05, ***P* < 0.01, ****P* < 0.001; **** *P* < 0.0001, ns = not significant. BMDMs, bone marrow-derived macrophages; *Lyz2*^cre/+^*Piezo1*^flox/flox^, conditionally Piezo1-deficient mice.

**
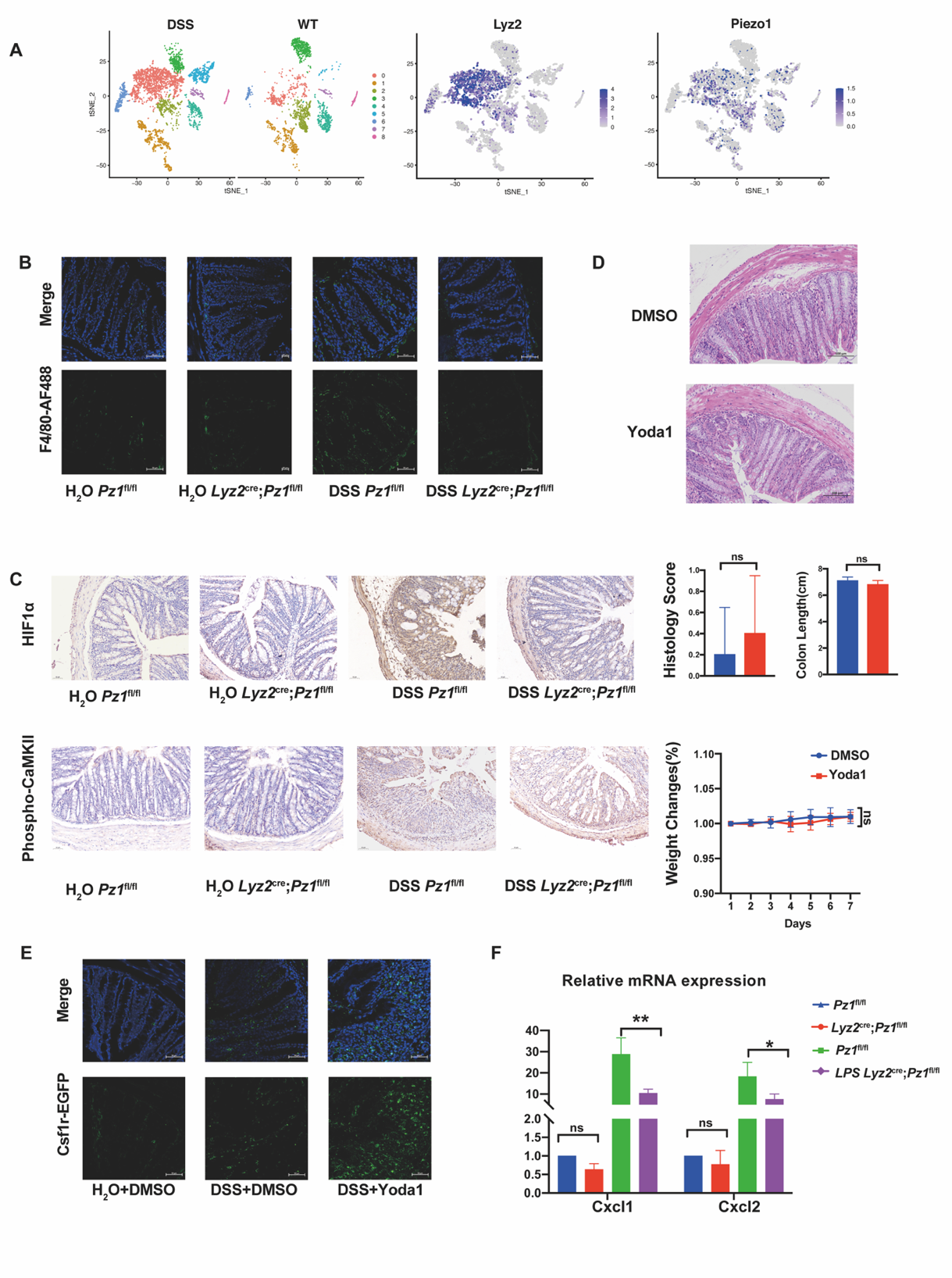
**

**Supplementary Figure 4.** (**A**) t-SNE plot and gene expression of myeloid gene clusters harvested from DSS mice colon analyzed by scRNA-seq. (**B**) Immunofluorescence of F4/80 expression in colons of *Lyz2*^cre/+^*Piezo1*^flox/flox^ mice and *Piezo1*^flox/flox^ control mice treated with DSS or H_2_O for 7 days. (**C**) Immunohistochemical figures of HIF1α and phospho-CaMKII in colons of *Lyz2*^cre/+^*Piezo1*^flox/flox^ mice and *Piezo1*^flox/flox^ control mice treated with DSS or H_2_O for 7 days. (D) Representative hematoxylin and eosin (H&E)-stained colon cross-sections, histological score, colon length and weight changes of mice treated with H_2_O for 7 days, to which 0.4 mg/kg Yoda1 or DMSO were added on days 1 and 4 (n=5). (E) Immunofluorescence of Csf1r expression in the colons of mice treated with DSS or H2O for 7 days, to which 0.4 mg/kg Yoda1 or DMSO were added on days 1 and 4. (F) Relative mRNA expression of Cxcl1 and Cxcl2 in BMDMs from *Lyz2*^cre/+^*Piezo1*^flox/flox^ and *Piezo1*^flox/flox^ control mice treated with or without 10ng/ml LPS (n=3). Statistical significances were calculated with two-tailed Student t test or one-way ANOVA, Tukey’s multiple comparisons test. Data are expressed as mean ± SD. **P* < 0.05, ***P* < 0.01, ****P* < 0.001; **** *P* < 0.0001, ns = not significant. BMDMs, bone marrow-derived macrophages; *Lyz2*^cre/+^*Piezo1*^flox/flox^, conditionally Piezo1-deficient mice.

| Target Gene | Forward Primer | Reverse Primer |
| --- | --- | --- |
| Glut1 | GGATCCCAGCAGCAAGAAG | CCAGTGTTATAGCCGAACTGC |
| Hk2 | TGATCGCCTGCTTATTCACGG | AACCGCCTAGAAATCTCCAGA |
| Hk3 | TGCTGCCCACATACGTGAG | GCCTGTCAGTGTTACCCACAA |
| Gpi1 | TCAAGCTGCGCGAACTTTTTG | GGTTCTTGGAGTAGTCCACCAG |
| Pfkl | GGAGGCGAGAACATCAAGCC | CGGCCTTCCCTCGTAGTGA |
| Pfkfb3 | CAACTCCCCAACCGTGATTGT | TGAGGTAGCGAGTCAGCTTCT |
| Aldoa | CGTGTGAATCCCTGCATTGG | CAGCCCCTGGGTAGTTGTC |
| Aldoc | AGAAGGAGTTGTCGGATATTGCT | TTCTCCACCCCAATTTGGCTC |
| Tpi1 | AAACCAAGGTCATCGCAGATA | CCCGGAGCTTCTCGTGTA |
| Gapdh | AGGTCGGTGTGAACGGATTTG | TGTAGACCATGTAGTTGAGGTCA |
| Pgk1 | CTGTGGTACTGAGAGCAGCAAGA | CAGGACCATTCCAAACAATCTG |
| Pgam1 | GCTGTGGTGTGACCAATGAA | TGACTATGCCCCCAGTTACC |
| Eno1 | GCCCTAGAACTCCGAGACAA | CAGAGCAGGCGCAATAGTT |
| Pkm | GCCGCCTGGACATTGACTC | CCATGAGAGAAATTCAGCCGAG |
| Ldha | CATTGTCAAGTACAGTCCACACT | TTCCAATTACTCGGTTTTTGGGA |
| HIF1α | TGAGTTCTGAACGTCGAAAAGA | CGGCATCCAGAAGTTTTCTC |
| β-Actin | CATGTACGTTGCTATCCAGGC | CTCCTTAATGTCACGCACGAT |
| Piezo1 | GTTACCCCCTGGGAACATCT | TTCAGGAGAGAGGTGGCTGT |
| Piezo2 | CTCACCTTTCCTGGCGTCAT | CCTCTTGAAACTCAGGCAGT |
| Trpv2 | TTAAATGACTTGTGAGGGAGATAGC | CAAGTAACACAATCTACCCAAGGTC |
| Trpv4 | TCACCTTCGTGCTCCTGTTG | AGATGTGCTTGCTCTCCTTG |
| Trpc1 | GCCATCTTTGTCACCAGGTT | GCTCGAGCAAACTTCCATTC |
| Trpc6 | GCAGGATTTCGTTGTTGGT | TGCTGACAGTTGGATGAGC |
| siHIF1α | CCAUGUGACCAUGAGGAAATT | UUUCCUCAUGGUCACAUGGTT |
| siNC | UUCUCCGAACGUGUCACGUTT | ACGUGACACGUUCGGAGAATT |

**Supplementary Table 1.** Allele-specific primers used in RT-qPCR

Inflammatory cell infiltrate Score1 Intestinal architecture Score2

Severity Extent Epithelial changes Mucosal architecture

Mild Mucosa 1 Focal erosions 1

Moderate Mucosa and submucosa 2 Erosions ±Focal ulcerations 2

Marked Transmural 3 Extended ulcerations ± granulation tissue ± pseudopolyps 3

Sum of scores 1 and 2 0-6

**Supplementary Table 2.** Histological score system
